# Update on Vertigo in Autoimmune Disorders, from Diagnosis to Treatment

**DOI:** 10.1155/2018/5072582

**Published:** 2018-09-26

**Authors:** Laura Girasoli, Diego Cazzador, Roberto Padoan, Ennio Nardello, Mara Felicetti, Elisabetta Zanoletti, Franco Schiavon, Roberto Bovo

**Affiliations:** ^1^Department of Neurosciences, Otorhinolaryngology Unit, University of Padua, Via Giustiniani, 2, 35128 Padova, Italy; ^2^Department of Medicine DIMED, Operative Unit of Rheumatology, University of Padua, Via Giustiniani, 2, 35128 Padova, Italy

## Abstract

The prevalence of autoimmune diseases has been increasing over the last 20 years. The clinical presentation of this large and heterogeneous group of disorders depends on whether the involvement is organ-specific or non-organ-specific. Dizziness, vertigo, and disequilibrium are common symptoms reported by patients with vestibulocochlear involvement. The association of vertigo and autoimmune diseases has been largely documented, suggesting that autoimmune disorders could be overrepresented in patients with vertigo in comparison to the general population. The aim of this review is to present the recent literature findings in the field of autoimmune-mediated diseases with cochleovestibular involvement, focusing on the clinical presentation, diagnosis, and treatment of immune-mediated inner ear diseases including autoimmune inner ear disease (AIED), Meniere's disease, and bilateral vestibulopathy, as well as of systemic autoimmune diseases with audiovestibular disorders, namely, Behçet's disease, Cogan's syndrome, sarcoidosis, autoimmune thyroid disease, Vogt-Koyanagi-Harada syndrome, relapsing polychondritis, systemic lupus erythematosus, antiphospholipid syndrome, IgG4-related disease, and ANCA-associated vasculitides.

## 1. Introduction

The percentage of autoimmune disorders in western countries is around 8% of the total population, though many studies reported an increase in prevalence and incidence over the last 20 years. The cochlear-vestibular system might be affected by autoimmune diseases although the diagnosis of autoimmune vestibular disorders is probably overlooked due to an absence of a reliable test that could identify the specific inner ear antigen [1].

Both sensory organs of the inner ear can be involved in autoimmune disorders: cochlea for the hearing and vestibular system for the balance. These two organs share the same sensory receptor, the ciliated cell, a mechanoreceptor with spontaneous activity able to signal not only the intensity and duration of a stimulus but also the direction in which it is applied. The apical surface of a ciliated cell is the mechanically sensitive portion. The basolateral portion is able to activate the terminations of the afferent nerve fibers, by releasing a neurotransmitter (glutamate). The nerve fibers in contact with the basal pole of the ciliate cell then convert the chemical stimulus into a discharge of action potentials that is sent to the central nervous system.

The sensory organs of the vestibular system are represented by the utricle, the saccule, and the three semicircular canals; they can perceive the gravity vector, the position of the head, and the linear, torsional, and angular accelerations that the head undergoes.

Variations in the spontaneous activity of the labyrinthine organs therefore provoke problems of equilibrium, ranging from modest sensations of instability, up to severe attacks of vertigo. Clinical features include dizziness, generalized imbalance, ataxia, motion intolerance, positional vertigo, oscillopsia, and episodic vertigo.

The objective of the present study is to provide an update on autoimmune disorders and audiovestibular consequences, especially for balance.

The review was conducted searching the relevant literature in the PubMed database for vertigo in autoimmune disorders. Works not focused on aspects of interest in the vestibulocochlear system were excluded.

Autoimmune vestibular disorders can be classified into two main groups: isolated immune-mediated inner ear disorders and audiovestibular pathology associated with autoimmune systemic manifestations.

## 2. Immune-Mediated Inner Ear Diseases

### 2.1. Autoimmune Inner Ear Disease (AIED)

Autoimmune disease of the inner ear is part of the large group of neurosensory hearing losses, of which it represents less than 1%. The disease is defined as a rapidly progressive, often fluctuating bilateral sensorineural hearing loss, which evolves over a period of weeks or months and which initially responds to immunosuppressive therapy [2]. Vestibular disorders coexist in 50% of patients and tinnitus in 25%, and clinical outcomes may be very similar to Menière's disease [3]. In 80% of cases, it is presented as bilateral, even if the involvement of the second ear can occur after months or years. Sensorineural hearing loss should be at least 30 dB, with evidence of progression in at least one ear in two successive audiograms performed over 3 months [4]. This is a rare condition with an incidence of about 5 cases per 100,000 people per year [5]. The prevalence in women and in the age group between 20 and 50 is higher. It may be associated in 15 to 30% of cases with a systemic autoimmune disease [6].

The concept of a role of immunity in idiopathic hypoacusia was introduced at the beginning of the century with the first experiments by Joannovic [7] and Masugi and Tomizuka [8] in the 1920s and 1930s. In 1958, Lehnhardt theorized the presence of “anticancer antibodies” as a cause of progressive bilateral hypacusia in 13 patients observed in his studies [9]. In analogy to sympathetic ophthalmopathy, Kikuchi hypothesized that the “sympathetic otitis,” i.e., the sensorineural hearing loss following surgery in the contralateral ear, had an autoimmune origin [10]. The German researcher Beickert was among the first to create an animal model, immunizing guinea pigs against tissues of the inner ear, verifying the subsequent appearance of cochlear damage, but not the hearing loss [11]. McCabe gave the definition of AIED after the observation of the beneficial effects of cortisone therapy on a series of 18 patients: “AIED is a progressively bilateral sensorineural hearing loss, that responds to the administration of immunosuppressives” [2]. Hughes et al. subsequently completed the definition of McCabe and introduced the distinction between primary or secondary autoimmune hearing loss, i.e., associated with a systemic autoimmune disease [12].

It has been hypothesized that the pathogenesis of this disease is linked to self-aggression by T lymphocytes against specific antigens of the inner ear with the formation of circulating autoantibodies. However, the demonstration of autoantibodies against internal ear antigens has been very difficult in AIED for three main reasons: the lack of specificity of autoantibodies in affected patients, the very low percentage of circulating autoantibodies, and the impossibility of performing diagnostic biopsies of the tissues involved in human in vivo. Due to the impossibility of having direct evidence with the induction of disease in humans, according to the Witebsky postulates, the diagnosis of autoimmune disease in AIED is mainly based on indirect tests in animal models (autoantibodies and autoreactive T lymphocytes) and on circumstantial evidence, such as association with other autoimmune diseases, lymphocyte infiltration of the cochlea, genetic correlation, and response to immunosuppressive therapy. There are numerous autoantibodies studied in the pathophysiology of AIED, some of the ubiquitous type, for example, HSP70 and collagen type II, others specific for the antigens of the inner ear (cochlina, *β*-tectorin, connexin 26, etc). Cochlina is mainly located in the spiral ligament and is the major component of the extracellular matrix of the inner ear after collagen; *β*-tectorin is present in ciliated cells, in support cells, and in the tectoral membrane. Activated T lymphocytes can enter the cochlea, exercising immunological surveillance. The transfer within the cochlea of CD4^+^ T lymphocytes activated with cochlina and *β*-tectorin antigens causes hypoacusia in the mouse model [13]. Immunohistochemical analysis with CD45^+^ shows leukocyte infiltration at the level of the inferior spiral ligament (5 weeks after immunization with Coch 131–150); activated T lymphocytes (INF-*γ* producers), both CD4^+^ and CD8^+^, are involved in cochlina recognition [14]. The deposition of immune complexes in the vascular stria causes damage to the capillary epithelium with alterations in vascular permeability that can cause endolymphatic hydrops, destruction of hair cells, collapse of the Reissner membrane, and membrane tectoria distortion [15]. In the animal model, there is a progressive fusion of the capillaries of the vascular stria, with the presence of perivascular inclusion at the level of the cochlear modulus [16].

The inner ear has always been considered a privileged immunological site: both for the absence of lymphatic drainage [17] and for the presence of an effective hemato-labyrinthine barrier. The hemato-labyrinthine barrier in the vascular stria is a highly specialized network that controls the exchanges between blood and interstitial space in the cochlea, necessary to maintaining the ionic gradient and the endocochlear potential for the active processes of mechanical-electrical transduction of the ciliate cells. Endothelial cells are connected by tight junctions, which together with the basement membrane form a barrier to the passage of many substances; the barrier is also formed by pericitis and perivascular melanocytes (the latter are activated during exposure to noise). Local immunity is regulated by the endolymphatic sac, the main antigen-processing site; its destruction causes a reduction of the immune response of the inner ear [18]. Other studies have subsequently shown the presence of immunoreactive cells in other areas of the inner ear, even in the absence of pathology. The migration of cells of hematopoietic origin, in particular of macrophages, has been described at the level of the modulus, of the lateral wall of the cochlea, and of the spiral ligament [19]. The cytokine cascade also activates the macrophages residing in the cochlea.

The diagnosis of AIED is still problematic as there are no reliable specific tests: it is mainly based on clinical evaluation and experience and must be suspected whenever we are evaluating a patient with a rapidly progressive idiopathic sensorineural hearing loss. García-Berrocal et al. have proposed the following criteria, which confirm the diagnosis of AIED in the presence of at least 3 major criteria, or 2 major and 2 minors [20]. Among these criteria, there is the rate of auditory recovery after corticosteroid or immunosuppressive therapy, which should be calculated as (Initial auditory threshold − final auditory threshold)/initial auditory threshold − auditory ear contralateral threshold) × 100(%).

Major criteria:

- Bilateral hearing loss
- Autoimmune systemic disease
- ANA title > 1 : 80
- Reduction of T-naive lymphocytes (CD4RA)
- Auditory recovery with therapy > 80%

Minor criteria:

- Unilateral hearing loss
- Young or middle-aged patients
- Female sex
- Auditory recovery with therapy < 80%

Otoscopy is generally normal, although lesions of the skin or cartilages of the outer ear may occur in relapsing polychondritis. Furthermore, lesions of the tympanic membrane, middle ear, and mastoid can be observed in Wegener's granulomatosis. Vestibular symptoms such as acute vertigo, disequilibrium, ataxia, and paroxysmal positional vertigo are present in 50% of patients. From 25 to 50% of patients also complain of tinnitus and auricular fullness. The differential diagnosis includes bilateral Menière disease, treatment with ototoxic drugs (gentamicin, cisplatin, etc.), enlarged vestibular aqueduct syndrome, Lyme disease, otosyphilis, toxoplasmosis, Charcot-Marie-Tooth disease, and intracranial hypertension [3].

Currently, no reliable diagnostic tests are available, although several studies have looked for a serological test applicable to patients with AIED [21]. In the mid-nineties, a 68 kDa protein was isolated from Western blot in the blood serum of some patients with AIED and Menière's disease, subsequently identified as an antibody to heat shock protein 70 (HSP70) [4]. The test was then marketed under the name of OTOblot, and its positivity as being predictive for a good response to steroid therapy was considered. Unfortunately, OTOblot proved to be a test with very low sensitivity and controversial specificity (Berrocal et al. [22]); subsequent studies have shown that the antibody binds the 68 kDa antigen of bovine cochlea, but not in humans, resulting in a ubiquitous, nonspecific ear protein. Probably the antigen target anti-68 kDa antibody is not HSP70 (as believed in the last 15 years) but choline transporter-like protein 2 (CTL2) [23]. For the possible correlation with systemic autoimmune diseases, a screening including antinucleus antibodies (ANA) was recommended; serum immunoglobulins IgG, IgA, and IgM; C3 and C4 complement factors; and immunophenotype of peripheral blood lymphocytes (PBL) [20].

Corticosteroid drugs remain the first-choice treatment for AIED, but immunosuppressive and immunomodulatory drugs can be used as adjuvant or second-choice treatments. Corticosteroids have an anti-inflammatory, immunosuppressive, and regulation mechanism in the electrolyte balance in the cochlea, thanks also to their mineralocorticoid action. The dosage and duration of therapy can be variable, and some protocols recommend 60 mg/day of prednisone (or 1 mg/kg/day) to be continued for at least 4 weeks; if a good hearing recovery is obtained, it is advised to continue with a maintenance dose for at least two months before a gradual reduction in dosages. On the other hand, if no auditory improvement is achieved after 4 weeks at full dosage, treatment is generally suspended. The response of patients with AIED, however, varies between 15 and 50%, and it is necessary to repeat a course of corticosteroids in case of relapses of hearing loss or vertigo [3]. Furthermore, the association of AIED with an autoimmune systemic disease generally requires the continuation of steroid therapy for at least 1 year. The limits of long-term high-dose steroid therapy are linked to the side effects of the drug.

The use of immunosuppressive drugs in AIED is described in the literature, including methotrexate, cyclophosphamide, and azathioprine. Methotrexate in the 1990s was used as a second-choice therapy in cases of poor hearing loss after steroid therapy; a multicentric randomized double-blind and case-control study published in 2003, however, showed that methotrexate is no more effective than placebo in hearing loss in these patients [24]. Cyclophosphamide remains an effective but limited therapeutic alternative with important side effects: pancytopenia, infections, hemorrhagic cystitis, bladder cancer, and infertility.

The advent of biologic drugs represented progress in AIED therapy: tumor necrosis factor (TNF) inhibitors showed good efficacy and little side effects. Their action effect is rapid (from hours to days) and is contraindicated only in the presence of active infections. Infliximab (Remicade) would have demonstrated a good effectiveness when applied in the forms of AIED associated with other organ diseases [25].

Etanercept (Enbrel) has been used in AIEDs with controversial results [26]. Several years' research is underway for the intracochlear administration of drugs or other substances, using different technologies. Micropumps with endocochlear catheters, nanoparticles, and viral vectors are being tested [27]. Recently, the use of vectors consisting of genetically modified monocytes/macrophages has been hypothesized. This hypothesis of gene therapy is based on the ability of circulating monocytes and macrophages to be recruited into the inner ear in a condition of inflammation. These “intracochlear drug delivery” techniques would allow a high and generally well-controlled concentration of the drug in the inner ear, reducing its systemic effects. On these topics, careful in vivo experiments will be required before clinical use in the AIED [27]. There are also methods of intracochlear administration that take advantage of the permeability of the round window, that is, the intratympanic injection or insertion of drugs soaked in the fenestral region after the surgical lifting of the tympanic membrane under local anaesthesia. These methods have been described since the 40s of the last century, unfortunately with generally modest and contrasting results.

If AIED does not respond to immunosuppressive therapy, the evolution of hearing loss must be carefully monitored with the serious execution of an internal ear MRI. In fact, fibrosis and ossification of the cochlea can evolve rapidly even within a few weeks and only the loss of the hyperintensity of labyrinthine liquids in the sequences in T2, or the image of initial ossification to the CT, allows the timely recognition of these processes. In these cases, the application of a cochlear implant must absolutely take place before fibrosis prevents the insertion of the electrode cable into the cochlea. The correct timing of the cochlear implant is a delicate decision that requires close cooperation between the ear surgeon, rheumatologist, and radiologist. A delay in the recognition of cochlear fibrosis may permanently preclude the rehabilitation of deep deafness or cophosis [28].

### 2.2. Menière's Disease

Menière's disease (MD) is a clinical disorder defined as the idiopathic syndrome of endolymphatic hydrops and characterized by a triad of fluctuating vertigo, tinnitus, and sensorineural hearing loss (with aural fullness). The Committee guidelines of 1995 consist of a diagnostic scale which includes diagnoses of “definite MD,” “certain MD,” “possible MD,” and “probable MD.”

The exact aetiology and pathogenesis mechanism of MD is still unclear, although several etiologies have been proposed, as autoimmune, allergic, genetic, traumatic, or infectious (viral). Menière's disease is a syndrome certainly caused by multiple factors; nonetheless, autoimmunity has a significant role in MD, thought to represent less than 20% (6% of unilateral and 16% of bilateral forms). The role of autoimmunity as aetiology is supported by: high prevalence of systemic autoimmune diseases in patients with MD than in the general population [29], bilateral presentation of the disease in 25–40% of patients, good efficacy of glucocorticoids and anti-inflammatory treatments, and possibility of experimentally inducing hydrops by injection of antigens or monoclonal antibodies.

Furthermore, some studies confirmed elevated levels of autoantibodies or circulating immune complexes and antigen-antibody reactions, in the serum of MD patients and in animal inner ear tissues [30]. They have recently been identified in protoarray analysis-specific antigens that caused immune reactions with patient's serum, which can be good candidates for diagnostic biomarkers of MD [31].

Although most cases are sporadic, a familial form of the disease has been described, from 2.6% to 23.5% [32]. Familial MD (FMD) should be considered if at least two family members (first or second degree) fulfill all the criteria of definite or probable MD. Most families described have an autosomal dominant pattern of inheritance, but, as FMD shows clinical heterogeneity, mitochondrial and recessive inheritance patterns are also observed in some families. Linkage studies in FMD have found candidate loci at 5q14–15 in a German family [33] and 12p12.3 in a large Swedish family [34], but the genes were not identified. By exome sequencing, Requena et al. [35] have identified mutations in DTNA and FAM136A genes in an autosomal dominant Spanish family with MD segregating the phenotype in three women in consecutive generations. DTNA encodes alpha-dystrobrevin, a dystrophin-associated protein which interacts with transmembrane proteins and actin in the basolateral membranes of epithelial cells, and it is associated with tight junction reorganization [36].

Genetic factors can be one of the causes of autoimmunity or increased immune reaction in Menière's disease. Recently, the first gene locus associated with sporadic Menière disease has been identified using a genotyping array; this locus (6p21.33), through the nuclear factor kB (NF-*κ*B) and the TWEAK/Fn14 pathway, is involved in inflammation modulation of many autoimmune diseases. Potential sites of this inflammatory damage are the blood–brain barrier, the endolymphatic sac, the spiral ligament, and the reticular lamina in the neurosensory epithelium of the cochlea. Patients with this genotype have been shown to develop bilateral Menière disease through a mediated NF-*κ*B inflammatory response [37].

Delayed endolymphatic hydrops (DEH) is a secondary endolymphatic hydrops that usually occurs suffering from longstanding hearing loss in one ear. The hearing loss can be caused by various causes: idiopathic, infectious, traumatic, etc. Its classification is still controversial in the international literature: DEH is considered in most cases as a secondary form of Menière, while for some Japanese and German authors, it is a pathology distinct from MD.

DEH mostly occurs in the ipsilateral ear, with episodic vertigo, or less frequently in the contralateral ear, with fluctuating hearing loss and recurrent vertigo. Symptoms, diagnosis and therapy are identical to those of Ménière's disease. In a few families, both unilateral hearing loss and DEH can have a genetic aetiology, showing autosomal dominant transmission with incomplete penetrance [38].

### 2.3. Bilateral Vestibulopathy (BVP)

This disease was called in 1989 by Baloh as “idiopathic bilateral vestibulopathy” [39] and was recently defined in a consensus document by the Classification Committee of the Bárány Society [40]. The prevalence of BVP in adults is estimated to be 28/100,000 [41], and the mean age of onset is around 50–60 years; often, there is a diagnostic delay due to unclear symptoms and signs.

The BVP develops in most cases slowly and progressively; in the initial phase of the disease, patients can report recurrent short-term episodes of vertigo, with or without association of hearing loss. Both labyrinths and/or vestibular nerves can be affected, simultaneously or sequentially. The symptoms in BVP are caused by the sensory vestibular impairment leading to insufficent vestibulospinal reflexes. This disease has a negative impact on social and physical functions, with decay of the health-related quality of life in 90% of the patients.

The diagnosis is based on patient anamnesis (movement-dependent postural imbalance and unsteadiness of gait) and clinical finding (bilaterally reduced or absent function of the VOR). Symptoms are exacerbated in darkness and on uneven ground, because they depend more on visual and somatosensory control, and they can disappear under static conditions; some patients can present movement-induced oscillopsia, for example, during rapid head turns.

Diagnostic criteria for bilateral vestibulopathy (BVP) have been defined by the Classification Committee of the Bárány Society [40]:

- Chronic vestibular syndrome with the following symptoms: unsteadiness when walking or standing plus at least one of 2 or 3; movement-induced blurred vision or oscillopsia during walking or quick head/body movements; and worsening of unsteadiness in darkness and/or on uneven ground
- No symptoms while sitting or lying down under static conditions
- Bilaterally reduced or absent angular VOR function documented by bilaterally pathological horizontal angular VOR gain <0.6, measured by the video-HIT or scleral-coil technique, and/or reduced caloric response, and/or reduced horizontal angular VOR gain <0.1 upon sinusoidal stimulation on a rotatory chair and a phase lead >68 degrees
- Not better accounted for by another disease

The aetiology remained unknown in more than 70% of cases [42]. The most frequent causes of BVP include ototoxic drugs, bilateral Menière's disease, infections, autoimmune diseases, tumors, bilateral labyrinth contusion, and vascular abnormalities. A genetic cause can be identified as 15–25% of cases [43]. In 20% of patients, the BVP is associated with gangliopathy, described in 2011 as CANVAS (cerebellar ataxia, nonlength-dependent sensory neuropathy, vestibular areflexia) [44], while 50% of the idiopathic BVH patients present with migraine according the International Headache Society criteria [43]. In a recent paper, it was reported that 23.4% of patients with idiopathic BVP had autoimmune disorders in their medical history; autoimmune disorders might not always cause BVH directly, but it seems reasonable to admit that autoimmune response could have a modulating role in this disease.

Most patients presented with a mutation in their COCH gene. This mutation has been identified to cause autosomal dominant nonsyndromic hearing loss accompanied by vestibular disorders (DNFA9). Although a strong underlying familial character seems to be present in multiple vestibular disorders, genome-wide association studies remain very difficult to perform, partly due to the clinical heterogeneity of vestibular disorders [43].

## 3. Audiovestibular Pathology Associated with Systemic Autoimmune Diseases

### 3.1. Behçet's Disease

Behçet's disease (BD) is a rare systemic immune mediate disorder of unknown aetiology characterized by the presence of recurrent oral and genital ulcers, ocular inflammation, and skin lesions. The aetiology and pathogenesis of BD are unknown although epidemiological data suggest the involvement of both genetic and environmental factors. The presence of HLA-B 51 represents a significant predisposing genetic factor. BD can affect any age group, but its onset in early and advanced age is relatively rare, the most common age of presentation being around the third decade of life, with a balanced male/female ratio [45]. BD is a systemic leukocytoclastic vasculitis, and any part of the organism may be affected. The prevalence of vestibular lesions ranges from 15% to 47% in separate studies. Morales-Angulo et al. reported high-frequency sensorineural hearing loss, vertigo, and bilateral vestibular hypofunction, but also in one patient were symptoms compatible with vestibular neuritis as the first manifestation of central nervous system (CNS) involvement in the context of neuro-Behçet. Brama and Fainaru reported in a selected group of 17 patients with vestibular lesions the presence of dizziness (82%), spontaneous nystagmus (11%), abnormal saccades (5%), abnormal caloric test (29%), and alteration in the rotational tests (58%) [46, 47]. Kulahli et al. found that 47% of patients had audiovestibular symptoms, with 8% complaints of vertigo [48]. Endolymphatic hydrops, with a presentation similar to bilateral Menière disease (fluctuating hearing loss, severe hearing loss, and vertigo), has been also reported [49, 50].

Although a specific audiogram pattern of hearing loss does not exist, sensorineural high-frequency downward audiometric slopes are frequently reported in BD [51]. The diagnoses of BD can be delayed by years when audiovestibular impairment is the presenting symptom in the absence of classical signs and symptoms, as in the case of a 66-year-old man with peripheral bilateral vestibulopathy and severe sudden SNHL as disease onset, reported by White and colleagues [52]. BD enters in the differential diagnosis of acute vestibular syndromes accompanied by SNHL.

Vestibular function studies seem to identify a peripheral lesion more frequently than damage to the central vestibular tract, although other authors found also a higher prevalence of central vestibular syndrome [48, 53]. A possible relationship between age or the disease duration and inner ear involvement is still uncertain.

First defined in 1954, [54], central nervous system (CNS) involvement (neuro-Behçet) occurs in 5–10% of patients, more frequently in males, and it usually occurs around 5 years after the onset of the disease [55, 56].

Vertigo, sensorineural hearing loss, tinnitus, and imbalance may be the initial symptoms of the mild form of neuro-BD. Neuro-BD may be parenchymal (80% of patients), nonparenchymal, or mixed. Parenchymal brain disease affects the brain system and/or basal ganglia while nonparenchymal brain disease is characterized by cerebral venous thromboses, arterial vasculitis, and aseptic meningitis. Koçer et al. estimated that the pontobulbar region in NB is affected by focal lesions in 40% of cases and Lee et al. and Gan et al. reported clinical cases of relapsing vertigo due to multiple recurrent reversible occlusions of basilar artery and/or postero-inferior cerebellar artery (PICA) [57–59].

According to a recent review reporting 130 cases of neuro-Behçet's disease in the pediatric population, the median age at presentation was 12 years with a male gender prevalence. Vertigo and/or hearing loss were present in 5.6% of the patients and only in the parenchymal form of the disease, with a prevalence of 21.4% among this subgroup of patients [60]. Treatment with immunosuppressive therapies or antitumor necrosis factor drugs revealed good prognosis with recovery or significant improvement of symptoms [60, 61].

To date, there are no diagnostic tests, therefore diagnosis is based on clinical symptoms. But it could be challenging when the symptoms are not concomitant or when BD is characterized by episodes of relapses and remission. The ICBD criteria included recurrent oral and genital aphthosis, eye lesions (anterior or posterior uveitis, cells in vitreous on slit lamp examination, and retinal vasculitis), skin lesions (erythema nodosum, pseudofollicolitis or papulopustular lesions, and acneiform nodules), neurological manifestation, vascular manifestation, and/or a positive pathergy test [62]. Increase in inflammatory markers, peripheral leukocytosis, and moderate anemia of chronic disease may be present but are not specific.

Multiple treatments have been proposed for BD. The European League Against Rheumatism (EULAR) proposed general treatment recommendations for the management of BD, not specifically for inner ear involvement. High doses of corticosteroid and immunosuppressive drugs (cyclophosphamide, azathioprine, and methotrexate) are recommended in case of parenchymal brain involvement [63]. Recently, anti TNF-alpha inhibitors have demonstrated to improve neurological symptoms and parenchymal lesions in 94% of 17 patients with neuro-BD and a complete response was achieved in one-third of cases. The onset of action was fast as the median time to achieve remission was of 3 months [64].

### 3.2. Cogan's Syndrome

Cogan's syndrome (CS) is a rare chronic inflammatory disease, characterized by nonsyphilitic ocular keratitis and Mèniére-like cochleovestibular dysfunction, with relapsing attacks of vertigo, sudden onset of tinnitus, vomiting, and progressive, mostly bilateral sensorineural hearing loss [65]. The hearing loss is generally bilateral and progresses over a period of 1 to 3 months to complete deafness in 60% of cases [66]. Haynes et al. proposed the definition of “typical” CS, with the ocular inflammation in addition to cochleovestibular symptoms, and “atypical” CS characterized by (a) ocular lesions other than interstitial keratitis (conjunctivitis, scleritis, iritis, choroiditis, subconjunctival, or retinal hemorrhage), (b) a delay of more than 2 years from the onset of ocular and cochleovestibular symptoms, and (c) systemic manifestations, including cardiovascular, neurological, and gastrointestinal symptoms [67]. Atypical disease is associated with systemic vasculitis in 20% of cases [68]. Furthermore, life-threatening aortic insufficiency develops in 10% of cases.

The etiopathogenesis of CS is currently unknown, but some evidences suggest that it might be the result of an autoimmune process. Antibodies against a peptide antigen—called Cogan peptide—are found in the serum of patients affected by CS. This peptide showed similarity with autoantigen SSA/Ro and with Reovirus III major core protein lambda and shared sequence homology with the cell density-enhanced protein tyrosine phosphatase-1 (DEP1/CD148), which is expressed on the sensory epithelia of the inner ear and on endothelial cells [69]. Traditional antibodies such as antinuclear antibodies and rheumatoid factor are not consistently found, but in 15–30% of cases, especially in the atypical form [70]. Recently, Jung et al. observed a lymphocytic, neutrophilic, and histiocytic infiltrate in both basal turns of the cochlea, vestibular system, and surrounding tissue, with thrombosis and necrosis of the small vessel wall. The organ of Corti and the stria vascularis were necrotic, the perilymphatic and endolymphatic spaces contained fibrotic material, and there was loss of inner and outer hair cells throughout the cochlea [71].

Treatment of CS traditionally consists of systemic corticosteroids, with ocular symptoms being more responsive than cochleovestibular symptoms. When a high dose of steroids is required, or relapsing symptoms are present, a second line of treatment with immunosuppressive agents is usually added. Nevertheless, from 43.5% to 52% of CS patients become deaf [72] and cochlear implantation represents a valuable rescue surgical strategy before cochlear partial obliteration or ossification, in both adult and pediatric patients [28, 73]. However, anecdotical cases of cochleovestibular function recovery are reported [74].

Recently, several studies described a treatment strategy with biotechnological drugs (biologic therapy), such as TNF-alpha inhibitors, anti-CD20 and anti-IL-6 receptors, for patients' refractory to the first- and second-line drugs. Infliximab appears to be the more frequently used biologic drug in the therapy of CS. In all cases but one, the treatment was able to lead to some improvement or stabilization of hearing loss and corticosteroid tapering. Durtette et al. confirmed that patients receiving infliximab experienced significant improvement in vestibuloauditory signs, different from patients with steroids alone or DMARDs, while systemic and ocular signs usually improve with steroids alone [75]. However, complete remission is rarely reported.

### 3.3. Sarcoidosis

Sarcoidosis is a granulomatous disease of unknown aetiology that can involve several organs. Its prevalence ranges from 10 to 40 per 100,000, with a mortality of 1% to 5% [76].

Environmental and genetic factors have been demonstrated in the pathogenesis of the disease. Environmental factors could include infections such as M. tuberculosis and P. acnes as well as other agents such as silica or metal dusts [77]. The Th1 response produces cytokines including interferon-gamma and IL 2, 6, and 12, recruiting additional phagocytes and T cells leading to the aggregation of phagocytes into epithelioid cells and giant cells. The hallmark of sarcoidosis is the noncaseating granuloma, consisting of activated macrophages and CD4-helper T lymphocytes. Granulomas are the source of ACE, which is found to be elevated in 60% of patients [78].

Neurologic involvement could occur in 5% of all sarcoidosis patients. It rarely occurs at disease onset, since about 40% of patients with neurosarcoidosis (NS) have previously diagnosed sarcoidosis in another organ, most commonly the chest or anterior uvea [79, 80].

Most commonly, patients present with cranial neuropathies, with facial nerve palsy being the most frequent. Otolaryngologic manifestations are found in 10% of patients [81]. Ear involvement and vertigo are also rarely described. They are observed in the presence of vestibulocochlear nerve involvement, presenting, therefore, with sensorineural hearing loss in association with vertigo and ataxia [82].

A review of cochleovestibular involvement in 50 cases of NS evidenced the presence of mostly bilateral, mild to profound sensorineural hearing loss in 94% of the patients, whose onset was mostly sudden (48%) or rapidly progressive (42%). Details of vestibular impairment were recorded in 64% of the patients, including vertigo, dizziness, and benign paroxysmal positional vertigo. The authors found a 96% rate of vestibular function loss in the tested cases, with a prevalence of bilateral impairment (67%) [83].

Cranial neuropathies in neurosarcoidosis are likely to be the result of a sarcoid basal meningitis affecting the exit of the lower cranial nerves. However, enhancing lesions within the internal acoustic canals (IACs) or in the CPA are reported on MRI, with the appearance of nerve meninges inflammation [83]. There are no randomized controlled trials in the treatment of NS, but general consensus suggests a rapid treatment with corticosteroids. High-dose systemic corticosteroids were administered either orally (prednisone 1 mg/kg/day) or through a short course of intravenous pulse (methylprednisolone 1000 mg per day, intravenously, during 3 days), followed by slow tapering, expecting a response within six to eight weeks [78]. For patients with refractory disease, immunosuppressants are usually considered. A specific DMARD is often chosen based on cost, availability, and side effect profile. Anti-TNF*α* agents have shown encouraging results in the treatment of ocular sarcoidosis, but further controlled studies are needed to elucidate their role [84].

### 3.4. Autoimmune Thyroid Disease

Autoimmune thyroid disease (AITD) is very common, affecting 1–5% of the entire population [85]. The most common are Graves' disease (GD) and chronic autoimmune thyroiditis also known as Hashimoto's thyroiditis (HT), with similar pathogenic mechanisms [86]. In both disorders, autoantibodies against thyroid antigens, including thyroglobulin, thyroid peroxidase, and thyroid-stimulating hormone receptor, can be detected. The clinical manifestations of thyroid dysfunction can range from hyperthyroidism as observed in Graves' disease, to symptomatic hypothyroidism, occurring in some HT patients.

A relationship between Meniere's disease (MD) and thyroid disease was firstly postulated in 1964 by Tamura, who hypothesized a role of hypothyroidism in the pathogenesis and progression of endolymphatic hydrops [87]. A second hypothesis was proposed in 1988 by Evans, who found positive anti-thyroid-microsome antibodies in 17% MD patients [88]. A recent study comparing MD patients to age- and sex-matched subjects suffering from non-Ménière acute unilateral peripheral vestibulopathy as well as healthy controls showed a significantly higher proportion of positive anti-thyroid autoantibody levels in MD patients (38%) than in both the control groups (7% and 12%, respectively) [89]. These findings confirmed a relationship between thyroid disease and MD, without clarifying the role of thyroid dysfunction. Santosh and Rao demonstrated a higher incidence of thyroid dysfunction in MD patients as well as the subjective remission of symptoms in all hypothyroid MD patients following 12 weeks of L-thyroxine replacement therapy [90].

The association between autoimmune disorders and vertigo has raised the hypothesis that autoimmune mechanisms might be also involved in the pathogenesis of benign paroxysmal positional vertigo (BPPV). In 2000, Modugno et al. [91] reported that 34/70 patients with BPPV had high levels of anti-thyroid autoantibodies, in the absence of other risk factors for BPPV. In a subsequent study, 200 euthyroid patients affected by HT were evaluated, showing that 18% of them had signs of BPPV and postulated a link between HT and the vestibular disease, possibly related to anti-thyroid autoantibodies, in particular a mechanical stimulation by immune complexes and the possible coexistence of a microangioitis in the inner ear, in the context of an autoimmune multiorgan disease [92].

HT is frequently associated to other organ-specific autoimmune disorders such as celiac disease, type 1 diabetes mellitus, Addison's disease, pernicious anemia, multiple sclerosis, vitiligo, and dermatitis herpetiformis [93]. Moreover, the increasing number of reports suggesting a possible “systemic” effect of thyroid autoimmunity on various tissues, including inner ear, open new perspectives on the interpretation of some clinical manifestations of HT [94]. Several reports have demonstrated a negative, direct effect of autoimmunity on connective tissue in various organs [95].

In a recent study, Chiarella et al., demonstrated that vestibular alterations were observed in 50% of HT patients and there was a significant correlation between vestibular alterations and serum anti-thyroid peroxidase antibodies but not with thyroid-stimulating hormone levels [96]. They postulated that the association between vestibular lesions and HT was mainly related to the autoimmune response against thyroid-specific antigens and was not influenced by the thyroid functional status. This concept would also imply a morbidity related to extra-thyroidal effects of thyroid autoimmunity, besides thyroid hormone insufficiency.

Nowadays, literature identifies patients with MD or BPPV as potential candidates to develop HT and vice versa, while it remains unclear whether anti-thyroid peroxidase and/or anti-thyroglobulin autoantibodies per se could promote thyroid dysfunction.

### 3.5. Vogt-Koyanagi-Harada Syndrome

Vogt-Koyanagi-Harada (VKH) syndrome is a systemic granulomatous autoimmune disease that affects melanocyte-rich tissues, such as the eye, inner ear, meninges, skin, and hair [97]. Most studies have found that women were affected more frequently than men and the disease onset was in the second to fifth decades of life [98]. Among all cases of uveitis, it was estimated to represent approximately 7% in Japan, 1–4% in the United States, and 3% in Brazil ([99]; Ohno 1977).

It is characterized by an acute onset of bilateral blurred vision with hyperemia (acute uveitis) preceded by flu-like symptoms. After the uveitic stage, vertigo with hearing loss, alopecia or vitiligo, and signs of meningeal irritation, including severe headache, periocular pain headache, and pleocytosis in the cerebrospinal fluid may appear. Peripheral vestibular dysfunction, such as rotato-horizontal or horizontal nystagmus, were observed in the vast majority of these patients by Yoshimoto [100]. Furthermore, Tahara and Sekitani [101] reported that vestibular function tests resulted to be abnormal in 77% of the patients, while symptoms or signs of central nervous system involvement were rarely demonstrated. On the other hand, in a recent series of 24 patients described by Ondrey et al. [102], only one (4%) had vertigo; elevated pure-tone thresholds were prevalent in eight (33.3%) and two (8.3%) experienced tinnitus.

About 17–73% of patients may progress to recurrence or chronicity [103], often associated with rapid tapering of corticosteroids, and this could lead to more severe and frequent audiovestibular abnormalities.

The etiopathogenesis of VKH is currently unknown, but some evidences reported an autoimmune aggression against antigens associated with melanocytes in a genetically susceptible individual after a virus infection trigger. Sugita et al. described that T cells from peripheral blood and intraocular fluid from patients with VKHD cross-reacted with tyrosinase protein and with highly homologous cytomegalovirus-specific sequences [104]. As suggested by recent studies, the immune response is aimed at proteins associated with melanocytes, with involvement of a T cell-mediated immune process with a peptide-specific Th1 cytokine response [105]. Cellular and humoral autoimmunity against retinal components has also been demonstrated in patients with VKH, as well as anti-Ro/SSA reactivity, in a small percentage of patients [106].

The diagnosis of VKH is primarily based on clinical features. The cornerstone of treatment of VKH is prompt, high-dose systemic corticosteroids, administered either orally (prednisone 1–mg/kg per day) or through a short course of intravenous pulse (methylprednisolone 1000 mg per day, intravenously, for three days), followed by slow tapering of oral corticosteroids throughout a minimum of a 6-month period. When a high dose of steroids is required, or relapsing symptoms are present, a second line of treatment with immunosuppressive agents is usually added [107]. Case series demonstrating the efficacy of several other treatment modalities are found in the literature including biologics agents, such as infliximab and rituximab [108].

### 3.6. Relapsing Polychondritis

Relapsing polychondritis (RP) is a rare, multisystemic autoimmune disease characterized by recurrent episodes of inflammation of the cartilaginous structures. It involves predominantly ears, nose, joint, vessels, and the respiratory tract [109]. RP affects 1 in 1.4 million people per year in UK [110] with a peak of incidence in the fifth decade of life, but the disease has been described in children and very old people. The clinical course of RP is variable, ranging from minor symptoms to a rapidly progressive illness and can be potentially lethal [111]. Otology manifestation in RP is common, and the otolaryngologist evaluation is frequently requested for the first diagnosis. The most frequent otology presenting symptoms is auricular chondritis, which is specific of RP once a local disease or infection has been ruled out. Episodes of nasal chondritis can lead to a collapse of the nasal septum with saddle-nose deformity, and laryngo-tracheal involvement can cause hoarseness dyspnea and stridor and may lead to subglottic stenosis due to recurrent laryngeal inflammation and laryngomalacia [112]. Vestibular dysfunction is rare and documented unilaterally or bilaterally in 6–13% of patients, usually in combination with cochlear involvement [113, 114]. Vestibular symptoms and sensorineural involvement may be due to the result of vestibular structure inflammation associated with chondritis, destruction of the Eustachian tube, and endolymphatic hydrops or due to the vasculitic process in the vestibular or cochlear branch of the internal auditory artery [112, 115].

Aetiology and pathogenesis of RP are not known, but many evidences suggest an autoimmune response against a yet unknown cartilage immunogenic epitope of chondrocytes or extracellular cartilage matrix antigen. Autoantibodies against collagen type II are detected in 33% of RP patients but also against IX and XI or other cartilage proteins such as cartilage oligomeric matrix protein (COMP) and matrilin-1 [116]. The immune-mediated process is hypothetically induced by different noxae (trauma, toxin, or infectious agents) which lead to the exposure of connective tissue or cell membrane self-epitopes in genetically susceptible individuals. The subsequent inflammatory process determines cartilage matrix destruction by protease release from inflammatory cells and from chondrocytes undergoing apoptosis [117].

Diagnosis is usually challenging, since it is based on a set of clinical evidence and imaging studies, and presenting manifestations are highly variable. Although not validated, the McAdam's or the Michet's diagnostic criteria are useful in the clinical setting. The former considers audiovestibular damage as an item required for diagnosis [118]; the latter defines hearing loss and vestibular dysfunction as minor criteria [114].

Unfortunately, there are no laboratory features to establish the diagnosis of RP yet. Antinuclear antibodies can be detected in 5–20% of patients [119]. Both p-ANCA positivity and c-ANCA positivity sometimes without antibodies to myeloperoxidase (MPO) or proteinase 3 (PR3) have been described [120]. Also, rheumatoid factor and antiphospholipid antibodies have been reported in RP [121].

Because of lack of clinical trials, the treatment of RP remains empirical. Corticosteroids are the cornerstone of the therapy. In inner ear involvement, high doses are required although permanent sequelae are common. Once the disease has been controlled, corticosteroid therapy should be gradually reduced but there are no definitive guidelines indicating how long-maintenance therapy should be continued before attempting withdrawal. Immunosuppressive agents should be considered if a satisfactory clinical response to steroid is not achieved or if high dosages of steroids are required to keep the patient in stable clinical status. Cyclophosphamide, azathioprine cyclosporine, and methotrexate are the most commonly drugs used. There are several reports about the successful treatment with the TNF-alpha antagonist, while for other biological agents such as abatacept (blocking of CD28 costimulation), rituximab (anti-CD20 antibody), and tocilizumab (anti-Il-6 receptor antibody), results are variable and the number of treated patients is too small to allow definitive conclusion [122].

### 3.7. Systemic Lupus Erythematosus

Systemic lupus erythematosus (SLE) is a chronic autoimmune disease with multiorgan involvement and an incidence of 12.5–39.0 per 100,000 people, higher in women, and it is two to three times more prevalent in African people [123]. The most frequent age at onset is from 20 to 40 years.

SLE is a multifactorial disease with different aetiologies, including genetic alterations, inflammation, drugs, environmental factors, and interactions between the adaptive and innate immune systems. The hallmark of SLE is the production of autoantibodies that react with self-nuclear and cytoplasmic antigens and culminate in immunologic attacks, resulting in tissue inflammation and multiorgan damage [124]. T and B lymphocyte disorder plays a central role in this autoimmune dysfunction, with an intense polyclonal B cell activation, with a population shift towards immature B cells and defects of T cells in signalling, adhesion, costimulation, gene transcription, and alternative splicing [125]. Autoantibodies are directed toward antigens at the nuclear cell level (ANA); one of the most relevant antigens in SLE is double-stranded DNA. They are highly specific for SLE, being present in 70% of cases, whereas they appear in only 0.5% of people without SLE [126]. Other ANA that may occur in people with SLE are anti-U1 RNP (which also appears in systemic sclerosis and mixed connective tissue disease), SS-A (or anti-Ro), and SS-B (or anti-La, both of which are more common in Sjögren's syndrome).

As a systemic disease, SLE has protean clinical manifestations, involving different organs including the skin, kidney, neurologic system, and musculoskeletal system. The clinical course is highly variable among patients and may be characterized by periods of remissions and of chronic or acute relapses.

Recent studies have also shown involvement of the inner ear [127, 128]. At this level, there are several mechanisms that lead to damage to the inner ear: (1) humoral-type antibody attacks on inner ear antigens, (2) cell-mediated cytotoxic damage to cochlear and vestibular hair cells, and (3) immune-complex deposition in the microvessels of the inner ear [124].

The most common otologic symptom found in clinical studies in SLE patients is sensorineural hearing loss (SNHL) that may be either slowly progressive or acute, with a reported prevalence of 6% to 70% [129–131]. Other audiovestibular symptoms associated with SLE include tinnitus and vertigo. Tinnitus was associated with hearing loss and may have been a consequence of deafferentation. The vestibular system appears to be involved in SLE, although to a lesser extent; vertigo and dizziness have rarely been reported. Only few authors have described vertigo in patients with SLE, and, in all cases, this symptom was always associated with SNHL or tinnitus [132, 133].

In a recent meta-analysis, the most common pathological findings in temporal bone specimens of SLE patients were polymorphonuclear infiltration (31%) and vasculitis (27%), followed by fibro-osseous reaction (21%), new bone formation (17%), and granulation (4%). Stria vascularis atrophy (33%) and spiral ganglion degeneration (23%) were two other consistent findings [127].

Different treatments for SLE-related hearing disorders have been proposed, focusing on prevention, especially for slowly progressive hearing loss, and hearing restoration for cases of sudden hearing loss. Corticosteroid therapy is routinely used for sudden hearing loss and for the prevention of further worsening of progressive hearing loss in SLE patients. Other treatments reported in literature are plasmapheresis as reported by Kobayashi et al. [134], anticoagulant therapy in case of antiphospholipid antibodies [130], and cyclophosphamide for refractory patients [135].

### 3.8. Antiphospholipid Syndrome

Antiphospholipid syndrome (APS) is an acquired disorder characterized by the association of antiphospholipid antibodies with thrombosis and pregnancy morbidity and mortality. It can be divided into a primary or secondary antiphospholipid syndrome, depending on the absence or presence of other autoimmune diseases, in particular SLE [136]. The etiopathogenetic mechanism involves hypercoagulability, thus determining microthrombosis and subsequent clinical consequences related to the affected vessels.

There is an increasing interest in the literature in investigating the association between idiopathic sudden sensorineural hearing loss (SNHL) and the occurrence of elevated antiphospholipid antibodies title, but the investigation of an underlying APS is rarely contemplated. Moreover, to date, no strong associations are reported concerning vertigo and APS. In fact, only sparse case reports marginally described the occurrence of vertigo in patients suffering from SNHL, where elevated antiphospholipid antibody title was identified. The first report was the case of a 23-year-old primigravida diagnosed with pre-eclampsia and elevated IgG anticardiolipine antibody level, who presented profound bilateral SNHL and loss of balance, with bilaterally absent caloric response [137]. Vertigo was also reported in 47% of patients with progressive SNHL, where the rate of elevated anti-cardiolipin antibodies was 16% [138]. Finally, only one case describes transient vertigo in a patient with sudden unilateral hearing loss diagnosed with APS [139].

The concept of an antiphospholipid inner ear syndrome has been recently proposed, assessing an elevated title for anticardiolipin, anti-B2 glycoprotein, and lupus anticoagulant antibodies in 25% of patients with idiopathic progressive SNHL with or without vertigo. Unfortunately, specific results concerning vertigo are missing [140].

### 3.9. IgG4-Related Disease

IgG4-related disease (IgG4-RD) is an idiopathic chronic relapsing-remitting inflammatory condition characterized by sclerotic pseudotumor formation in multiple organs, including head and neck structures, such as orbit, salivary glands, and thyroid [141]. The disease generally occurs most commonly in middle-aged and older men, but the epidemiology data requires further definition [142].

IgG4-RD can present as a single organ or a multiorgan disease; therefore, clinical manifestations could be extremely different. Due to their infiltrative nature and nonspecific radiologic findings, pseudotumors are often confused with neoplasms or localized infection; therefore, the biopsy with immunostaining is mandatory [143]. The pathogenesis is not completely known; up to date, the hypothesis identifies a T-follicular helper cell response responsible for the development of germinal centers within lymph nodes and involved organs and the production of cytokines (e.g., IL-4) that drive the IgG4 class switch, culminating in the creation of IgG4-secreting plasmablasts and long-lived plasma cells. This continuous antigen presentation by B cells and plasmablasts sustains a clonally expanded population of CD4^+^ cytotoxic T lymphocytes, which produces potentially important mediators of the fibrosis. [144].

Although the clinical presentation, diagnosis, and treatment for IgG4-RD involving head and neck have been described in the literature, there is a limited number of data regarding otologic manifestations and temporal bone involvement. There are few published case reports of unilateral temporal bone involvement causing single-sided progressive hearing loss and vestibular dysfunction [145, 146].

In a recent case series of 39 patients with confirmed diagnoses of IgG4-RD, 12.8% had otologic symptoms, namely, two cases of eosinophilic otitis media, two of otitis media with effusion, and one of SNHL were registered [147]. The latter case also developed vertigo at disease onset.

Vestibular impairment was described in other two cases of IgG4-RD with pseudotumor lesions of the temporal bone, in one case presenting bilaterally. Neuroimaging techniques are mandatory to defining the extent of the disease inside and outside the temporal bone. Bone erosion of the labyrinth was observed, as well as pachymeningeal enhancement of the posterior fossa [148, 149].

Moreover, there is also evidence of a case of IgG4-related hypertrophic pachymeningitis of the posterior cranial fossa in a 52-year-old man presenting with vertigo, moderate bilateral hearing loss, tinnitus, and blurred vision. Initial disease progression under corticosteroid and immunosuppressive drug improved only after rituximab, leading to tinnitus and vertigo improvement within 6 months [150].

The typical histopathologic finding is a lymphoplasmacytic infiltrate, eosinophils, storiform fibrosis, and obliterative phlebitis and by immunohistochemistry demonstrating IgG4-positive plasma cells >10 per HPF and a ratio of IgG4/IgG^+^ cells >40% [151]. However, histological diagnosis is difficult using specimens from the middle ear and it is recommended that biopsy specimens should be taken from the paranasal sinus or nose. Serum IgG4 levels may be elevated; however, these are normal in 30% to 50% of patients with IgG4-RD [152].

Steroids are often used as induction therapy or as adjunctive therapy to surgery for symptomatic IgG4-RD. Most of the patients respond to steroids within several weeks, particularly in early stages of the disease. In refractory cases, immunosuppressive drugs should be considered. Rituximab, a monoclonal antibody against the short-lived CD20^+^ B cells, has been demonstrated to be an effective treatment [153]. Surgical resection with adjunctive corticosteroids may be required for temporal bone IgG4-RD, which has been suggested to follow a more aggressive course. In all reported cases of unilateral temporal bone IgG4-RD, tumor size reduction and disease remission were achieved with a combination approach of mastoidectomy and medical management [148].

### 3.10. ANCA-Associated Vasculitides

The antineutrophil cytoplasm antibody- (ANCA-) associated vasculitides (AAV) are heterogeneous, multisystemic, autoimmune diseases of unknown aetiology characterized by necrotizing small and medium vessel vasculitis. AAV are associated with ANCA autoantibodies, mainly directed against anti-proteinase 3 (anti-PR3) or anti-myeloperoxidase (anti-MPO) [154]. In the group of AAV are listed granulomatosis with polyangiitis (GPA, formerly Wegener's granulomatosis), microscopic polyangiitis (MPA), and eosinophilic granulomatosis with polyangiitis (EGPA, previously known as Churg-Strauss syndrome).

GPA, MPA, and EGPA have respective annual incidence rates of 2.1–14.4, 2.4–10.1, and 0.5–3.7 per million in Europe, and they are slightly more common in men, typically those aged over 60 years [155].

MPO and PR3-specific ANCA can activate neutrophils and monocytes through their Fc receptors, which determines their adhesion to endothelial cells where degranulation occurs. This releases free oxygen radicals and lytic enzymes, resulting in damage to the endothelium via the induction of necrosis and apoptosis. Furthermore, neutrophils release chemoattractive signalling molecules that recruit more neutrophils to the endothelium, acting as a positive feedback loop [156]. In addition to this mechanism, the role of ANCA-reactive B cells has recently been proposed, which further leads to neutrophil degranulation and inflammation [157].

AAV may involve organs throughout the body, such as the eye, nose, ear, lung, and kidney, and the presentation could range from a skin rash to a fulminant multisystem disease [158]. Typical features of GPA include nasal crusting, discharge and epistaxis, uveitis, upper respiratory tract involvement, and often, when in the context of an active urinary sediment, renal involvement. Patients with MPA are typically older and present with more severe renal diseases than GPA together with rash and neuropathy. EGPA typically presents with a multisystem disease on a background of asthma, nasal polyposis, and peripheral blood eosinophilia.

It has been reported that 19 to 61% of systemic AAV patients show otologic symptoms such as otalgia, hearing loss, aural fullness, tinnitus, and vertigo during their clinical courses, and in turn, otologic symptoms are sometimes the initial manifestation of AAV [159, 160].

In GPA patients, Takagi et al. [161] described classification of otologic involvement into 5 distinct patterns: (1) serous otitis media, the most common (90%), resulting from Eustachian tube obstruction and nasopharyngeal involvement; (2) SNHL (43%) due to vasculitis of the cochlear vessels and the immunocomplex deposits in the cochlea; (3) chronic otitis media (24%) caused by middle ear mucosa lesions; (4) vertigo resulting from central system involvement and immunocomplex deposits in the vestibular portion of the inner ear; and (5) facial nerve palsy (8%) usually associated with otitis media, secondary to compression of the nerve in the middle ear course, especially in the presence of dehiscence in the fallopian canal. In a recent paper, Morita et al. [162] noted that the bilateral or at least the unilateral vestibular periphery was affected in 84% of the GPA patients with otologic involvement. The authors described two types of vestibular symptoms: chronic dizziness accompanied by progression of hearing loss and acute vertigo attack with sudden hearing loss that mimics sudden deafness. In the latter case, vertigo may be caused by acute dysfunction of the vestibular periphery, and it would be difficult to make a correct diagnosis of this condition at the early stage. Temporal bone histopathological studies in GPA patients revealed hemorrhages in the stroma of the crista of semicircular canals, changes in vessel caliber, and lymphocytic infiltration [163].

The most typical otological manifestation of EGPA is chronic granulomatous otitis with chronic, thick aural discharge, which is responsible for conductive hearing loss [164]. Middle ear involvement is a self-sustaining disease, with no direct correlation with the nasal counterpart. The pathogenic mechanism of the otitis media is probably due to eosinophilic infiltration, thick secretions, and mucosal edema that may block the functioning of the Eustachian tube, and consequently middle ear ventilation. However, the complete etiopathogenesis of otologic manifestations in EGPA is still unknown and may be multifactorial. In patients with sensorineural hearing loss but no signs of middle ear involvement, the presence of a vasculitic process causing an eight-cranial-nerve neuropathy could be hypnotized [165]. It remains not clear why the vasculitic process apparently spares the vestibular system, as demonstrated by the lower percentage of vestibular disorders compared to audiological problems.

Management of the otological complications of AAV is challenging. Medical therapy is the main treatment and is based on a steroid regimen, both systemic and local. Surgical treatment is generally limited to the application of a ventilation tube, which significantly increases audiological performances and the patient's quality of life. Other surgical treatments, such as tympanoplasty, are reserved for complicated cases.

Although immunosuppressive therapy in addition to steroid treatment may be effective for preventing progression to intractable dizziness, other variables such as general health, lifestyle, other systemic diseases, and genetics might also influence the recovery rate. [162].

## 4. Conclusions

Autoimmune vestibular disorders are rare and probably underestimated diseases in the general population.

Currently, there are no specific diagnostic tests able to identify the presence of autoimmune or immune-mediated diseases in the inner ear. Therefore, classification systems and diagnostic criteria including the ENT involvement for systemic autoimmune diseases are of utmost importance.

Physicians should be aware of cochleovestibular symptoms every time he suspects an autoimmune disease, with specific oriented questions in the anamnesis in order to investigate the presence of hearing or balance problems. On the other hand, ENT specialists need to perform a complete clinical-instrumental evaluation every time he exams a patient with a certain or suspected history of autoimmune disease; the hearing test and the vestibular bed-side examination are mandatory, and if the condition required further investigation, it is advisable to perform specific vestibular tests, such as electronystagmography, posturography, and VEMPs.

A close collaboration between rheumatologists, radiologists, and otolaryngologists is essential to recognizing patients with indication to systemic therapy.

Timely and adequate medical treatments allow recovery of audiovestibular damage in most cases. However, the evolution of hearing loss and vertigo must be carefully monitored because fibrosis and ossification of the inner ear can evolve rapidly even within a few weeks.

The introduction of new classifications could lead to the use of effective immunological therapies, such as biological drugs, even in those cases limited to the posterior vestibule. New molecules and the above “intracochlear drug delivery” methods currently being tested will allow a more effective, personalized therapy with fewer side effects in the future.
